# Towards conserving natural diversity: A biotic inventory by observations, specimens, DNA barcoding and high-throughput sequencing methods

**DOI:** 10.3897/BDJ.8.e50124

**Published:** 2020-02-27

**Authors:** Matthew Lewis Bowser, Rebekah Brassfield, Annie Dziergowski, Todd Eskelin, Jennifer Hester, Dawn Robin Magness, Mariah McInnis, Tracy Melvin, John M. Morton, Joel Stone

**Affiliations:** 1 U.S. Fish & Wildlife Service, Kenai National Wildlife Refuge, Soldotna, Alaska, United States of America U.S. Fish & Wildlife Service, Kenai National Wildlife Refuge Soldotna, Alaska United States of America; 2 Salish Kootenai College, Pablo, Montana, United States of America Salish Kootenai College Pablo, Montana United States of America; 3 U.S. Fish & Wildlife Service, North Florida Ecological Services Office, Jacksonville, Florida, United States of America U.S. Fish & Wildlife Service, North Florida Ecological Services Office Jacksonville, Florida United States of America; 4 City of Soldotna, Planning and Zoning Commision, Soldotna, Alaska, United States of America City of Soldotna, Planning and Zoning Commision Soldotna, Alaska United States of America; 5 Auburn University, School of Forestry & Wildlife Sciences, Auburn, Alabama, United States of America Auburn University, School of Forestry & Wildlife Sciences Auburn, Alabama United States of America; 6 Michigan State University, College of Agriculture & Natural Resources, Department of Fisheries and Wildlife, East Lansing, Michigan, United States of America Michigan State University, College of Agriculture & Natural Resources, Department of Fisheries and Wildlife East Lansing, Michigan United States of America; 7 U.S. Fish & Wildlife Service (retired), Soldotna, Alaska, United States of America U.S. Fish & Wildlife Service (retired) Soldotna, Alaska United States of America; 8 University of Alaska Fairbanks, Fairbanks, Alaska, United States of America University of Alaska Fairbanks Fairbanks, Alaska United States of America

**Keywords:** biomonitoring, metabarcoding, vegetation, birds, terrestrial invertebrates, earthworms

## Abstract

The Kenai National Wildlife Refuge has been given a broad conservation mandate to conserve natural diversity. A prerequisite for fulfilling this purpose is to be able to identify the species and communities that make up that biodiversity.

We tested a set of varied methods for inventory and monitoring of plants, birds and terrestrial invertebrates on a grid of 40 sites in a 938 ha study area in the Slikok Creek watershed, Kenai Peninsula, Alaska. We sampled plants and lichens through observation and specimen-based methods. We surveyed birds using bird call surveys on variable circular plots. We sampled terrestrial arthropods by sweep net sampling, processing samples with High Throughput Sequencing methods. We surveyed for earthworms, using the hot mustard extraction method and identified worm specimens by morphology and DNA barcoding. We examined community membership using clustering methods and Nonmetric Multidimensional Scaling.

We documented a total of 4,764 occurrences of 984 species and molecular operational taxonomic units: 87 vascular plants, 51 mosses, 12 liverworts, 111 lichens, 43 vertebrates, 663 arthropods, 9 molluscs and 8 annelid worms. Amongst these records, 102 of the arthropod species appeared to be new records for Alaska. We found three non-native species: *Derocerasagreste* (Linnaeus, 1758) (Stylommatophora: Agriolimacidae), *Dendrobaenaoctaedra* (Savigny, 1826) (Crassiclitellata: Lumbricidae) and *Heterarthrusnemoratus* (Fallén, 1808) (Hymenoptera: Tenthredinidae). Both *D.octaedra* and *H.nemoratus* were found at sites distant from obvious human disturbance. The 40 sites were grouped into five community groups: upland mixed forest, black spruce forest, open deciduous forest, shrub-sedge bog and willow.

We demonstrated that, at least for a subset of species that could be detected using these methods, we were able to document current species distributions and assemblages in a way that could be efficiently repeated for the purposes of biomonitoring. While our methods could be improved and additional methods and groups could be added, our combination of techniques yielded a substantial portion of the data necessary for fulfilling Kenai National Wildlife Refuge's broad conservation purposes.

## Introduction

In order to conserve global biodiversity, given current and expected realities of species distribution shifts, novel assemblages and potential extinctions, we must be able to routinely document species distributions and assemblages. Historically, this has not been feasible except for a small set of large, easily-recognised species because of the taxonomic impediment—the difficulty, time and expense of identifying species from hyperdiverse groups (see Goldstein and DeSalle 2010).

High-Throughput Sequencing (HTS) methods have been advocated as a means of overcoming the taxonomic impediment, enabling identifications of species from diverse groups in mixed environmental samples (Hajibabaei et al. 2011, Baird and Hajibabaei 2012, Yu et al. 2012, Aylagas et al. 2016, Lobo et al. 2017, Porter and Hajibabaei 2018b, Bush et al. 2019, Watts et al. 2019). These HTS methods have recently been put into practice for biomonitoring of species assemblages of non-marine invertebrates (Gibson et al. 2015, Hajibabaei et al. 2016, Bush et al. 2019).

In this study, we tested the ability of several methods to rapidly determine species assemblages on a portion of a National Wildlife Refuge in Southcentral Alaska for fulfilling the broad conservation purposes of that refuge.

The United States Congress mandated that all Alaska National Wildlife Refuges must, "conserve fish and wildlife populations and habitats in their natural diversity," (96th Congress 1980); "provide for the conservation of fish, wildlife, and plants, and their habitats...," (105th Congress 1997); and, "ensure that ... biological integrity, diversity, and environmental health ... are maintained for the benefit of present and future generations..." (105th Congress 1997). Woodward and Beever (2010) noted that, under these broad purposes of National Wildlife Refuges in Alaska to protect natural landscapes and entire ecosystems, they "must develop a monitoring program to assess whether protective management is successfully conserving something as complex and open-ended as biodiversity."

Alaska Maritime National Wildlife Refuge and Kenai National Wildlife Refuge were given an additional purpose of providing opportunities for scientific research (96th Congress 1980).

Morton et al. (2009) recognised that a prerequisite for fulfilling the broad conservation mandates of Alaska National Wildlife Refuges was to document species distributions and assemblages. Given the remoteness of most of the land area of Alaskan Refuges, generally requiring access by aircraft and a need to obtain sample sizes large enough to make meaningful inferences about species distributions and communities over these land areas, they selected methods that would enable sampling of a large number of sites over a short time with minimal travel cost. Ensuring that all sampling methods could be executed in under 1 hr per visit enabled up to six remote sites to be sampled per crew per day.

These methods delivered meaningful metrics useful for accomplishing the Alaska National Wildlife Refuges' conservation and research purposes, but two key deficiencies were identified. First, the lack of spatially or temporally repeated sampling led to an inability to account for imperfect detection probabilities (as defined by MacKenzie et al. 2002, MacKenzie et al. 2005). Second, it was clear from the effort and time required to identify invertebrates using morphological methods that it would not be practical to repeatedly sample using these methods. In order to enable identifications of species by DNA barcoding, the U.S. Fish & Wildlife Service, Alaska Region supported development of a DNA barcode library of Alaskan terrestrial arthropods (Sikes et al. 2017a), setting the stage for biomonitoring using HTS methods.

In the current effort, we tested biomonitoring methods similar to those used by Morton et al. (2009), with the goal of maintaining efficient inventory and monitoring techniques for species assemblages, while addressing previous methodological shortcomings. We subsampled spatially to account for imperfect detection and we identified invertebrates using HTS methods. In this article, we describe our methods and the resulting biodiversity data. We intend to assess our ability to account for imperfect detection in a subsequent paper.

## Materials and methods

### Study area

Our study area comprised a section of the Slikok Creek watershed, a well-defined geographic region representative of the lowlands of the western Kenai Peninsula and the watershed. We determined the Slikok Creek watershed boundary (HUC12 code: 190203021804) using the national Watershed Boundary Dataset (U.S. Geological Survey 2016).

The 5,917 ha Slikok Creek watershed originates on the Kenai National Wildlife Refuge (KNWR) with headwaters in the wetlands and hills around Headquarters Lake, Nordic Lake and Slikok Lake. Streams from these lakes and wetlands coalesce into Slikok Creek, which then flows through a mosiac of public and private lands before joining the Kenai River.

**Figure 1.**
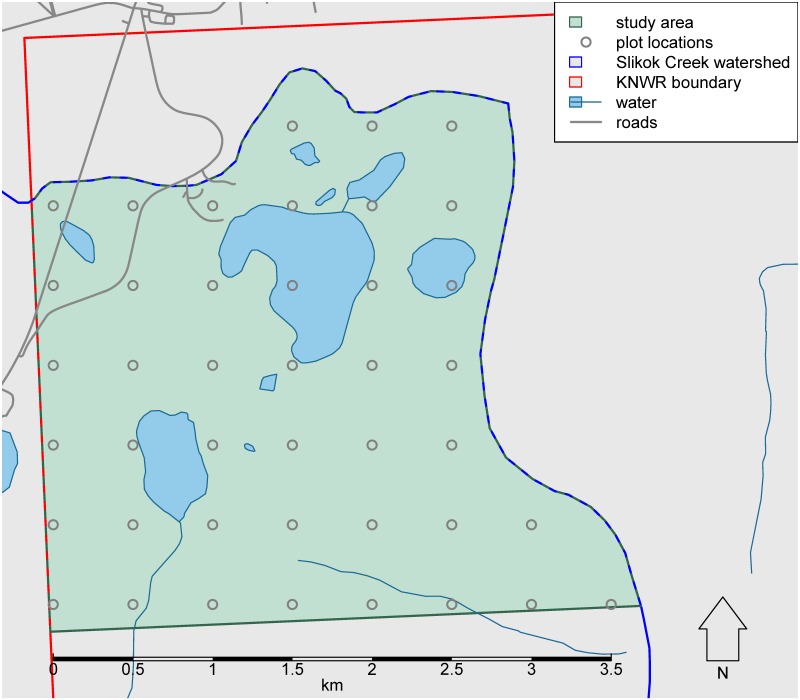
Map of the Slikok Creek watershed study area and sampling design.

We limited our study area to the part of the watershed within the KNWR. We also restricted our study area to the part of the watershed north of 60.44° latitude to eliminate long walking distances required to access the more remote southern portion of the watershed. This yielded a study area with boundaries not more than roughly 3 km from established roads and trails. The resulting 938 ha study area occupied a bounding box from 60.44° to 60.47° latitude and from -151.10° to -151.03° longitude (Fig. 1, Suppl. material 1).

### Sampling design

**Figure 2.**
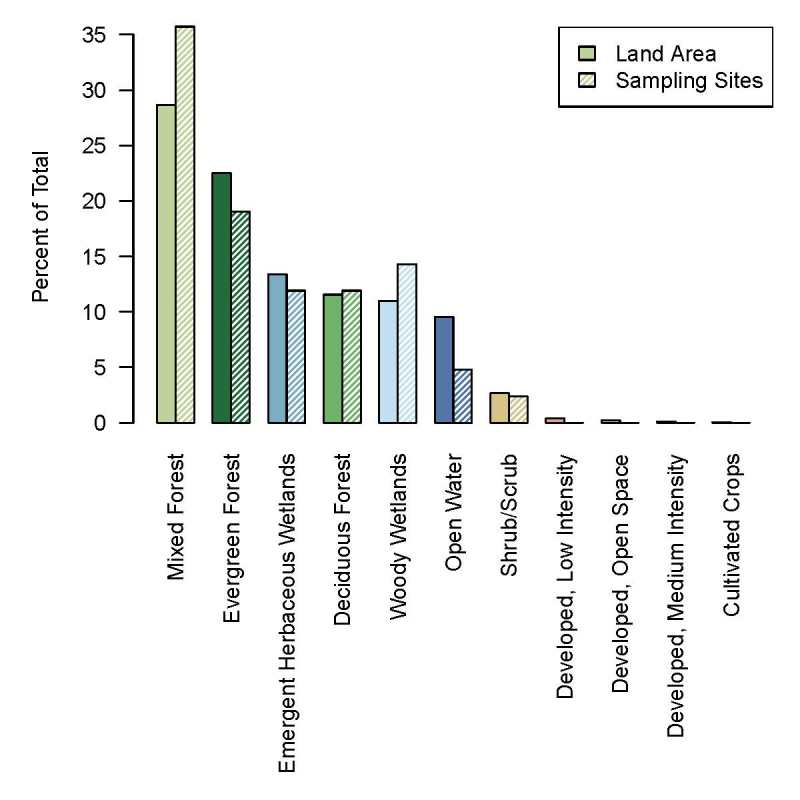
Breakdown of the study area and sample frame by cover classes. The cover classes and colours of the bars are those defined in the National Land Cover Database (NLCD).

For an initial field inventory, a 500 m grid was chosen by using the coordinates of the centroids of the 250 m pixels from the Alaska eMODIS product (Jenkerson et al. 2010), choosing every other centroid to make a grid of sites having 500 m spacing. The resulting sample frame consisted of 42 sites (Fig. 1, Suppl. material 1). This sample frame was representative of the study area in terms of proportions of cover classes (Fig. 2), based on cover classes from the National Land Cover Database (NLCD, Homer et al. 2015). For the present study, we excluded the two aquatic sites, leaving 40 terrestrial sites.

### Field methods

As in Morton et al. (2009), our interest was in methods that could be executed rapidly, requiring all sampling to take less than one hour per visit. This precluded consideration of any methods that would have required passive sampling (e.g. sound recording devices, camera traps, malaise traps, pitfall traps and pan traps).

#### Plot establishment

**Figure 3.**
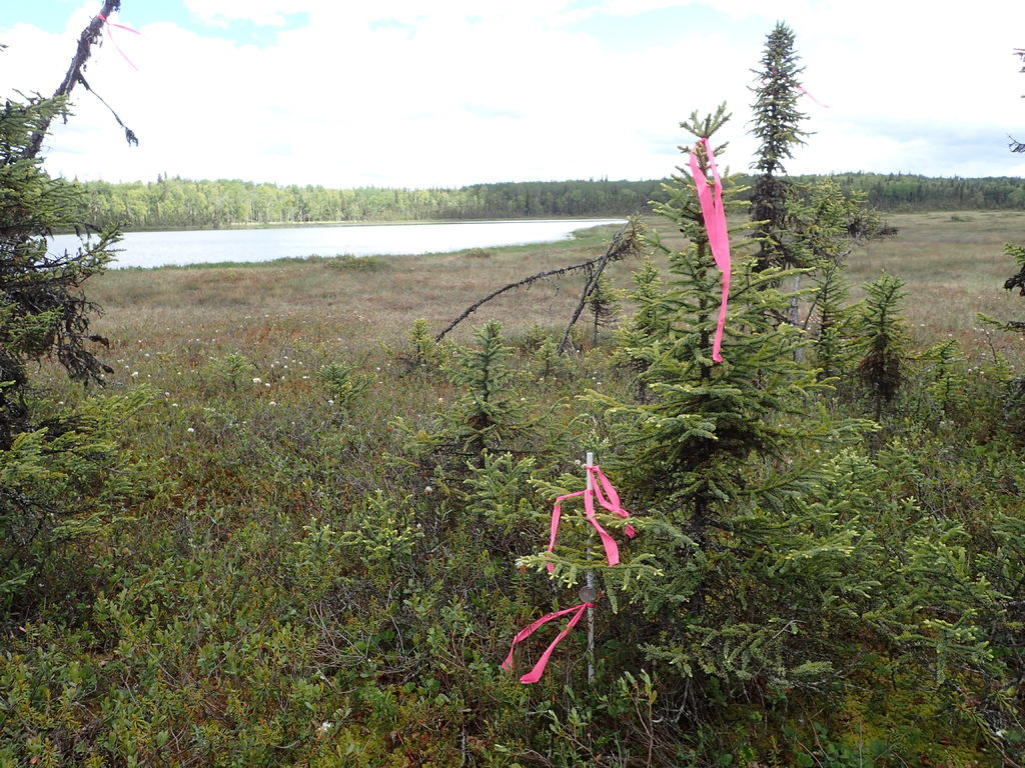
A sampling site marked with a fibreglass rod and temporary flagging (image details: https://doi.org/10.7299/X7F1901H).

Sampling sites were marked by driving 122 cm long, 13 mm diameter SunGUARD Smart Stake™ fibreglass rods into the ground, then labelling them with aluminium tags (Fig. 3). During the survey period, sites were also temporarily marked with high-visibility forestry flagging tape.

#### Plants and lichens

Vegetation was sampled from 18 July to 10 August 2016. We recorded presence within the 5.64 m radius circular plot and species identity of all vascular plants, bryophytes and lichens that could be identified in the field. In some cases, plants were collected from outside the plot to be identified in the lab. In addition, representatives of all bryophyte and lichen species present on the plots were collected from outside of the plot for subsequent identification. We collected from outside of plots to maintain integrity of the plots for the purpose of potential long-term monitoring.

#### Bird call surveys

Breeding bird calls were sampled from 14 June to 17 June 2016. We sampled bird abundance and occurrence using variable circular plot methods adapted from the Alaska Landbird Monitoring System (ALMS) protocol (Handel and Cady 2004, Morton et al. 2009), identifying birds in the field. Horizontal distances to each bird were estimated at 1 min increments during a 10 min sampling interval using auditory or visual cues. Surveys were conducted 30 min after sunrise during the first 4-5 h of the morning.

#### Earthworms

Earthworms were sampled concurrently with vegetation sampling, using the hot mustard extraction method (Lawrence and Bowers 2002). At each site, a quadrat was selected from the 5.64 m radius circular plot, but within about 20 m from the circular plot. Where available, we selected sites where ample hardwood leaf litter was available to increase the probability of detecting earthworms. A 0.5 m square (0.25 m^2^), metal frame was used as a quadrat. Within this quadrat, litter was first removed carefully by hand and all worms found were collected. Next, about 2 l of mustard powder mixture (30 g yellow mustard powder and 3.8 l of water) were poured evenly over the quadrat. Worms were collected for the next 5 min as they emerged from the soil, then the hot mustard extraction was repeated using the remaining mustard powder mixture.

#### Sweep net samples of terrestrial arthropods

Sweep net samples of terrestrial arthropods were collected concurrently with the bird surveys from 14 June to 17 June 2016. A second set of sweep net samples was collected when the plots were revisited to sample vegetation on 18 July to 9 August2016. A total of 160 sweep net samples were collected (40 plots × 2 samples/plot × 2 visits/plot).

Arthropods were sampled within a 100 m^2^, 5.64 m radius, circular plot using the centre stake as plot centre. To enable comparison with the previous work of Morton et al. (2009), we used the same methods except that we subsampled spatially. We split the plot into two subplots, dividing along the north-south axis. Each semicircular subplot was independently sweep-netted, such that the entire area was swept from the ground surface up to a height of roughly 2 m. No defined pattern of sweeping was enforced, but we ensured that all substrates and macrohabitats within reach were swept over once within a time limit of 5 min per sample. We used a BioQuip™ model 7112CP 30.5 cm diameter net with a BioQuip™ model 7312AA 30.5 cm extension handle and a BioQuip™ model 7112CPA net bag with a mesh size of approximately 8 × 9 meshes/mm.

All specimens were collected into a single Nalgene^®^ model 2104-0008 wide-mouth 250 ml bottle containing UniGard -100 propylene glycol antifreeze. Even though we could have used ethanol as a preservative, we chose propylene glycol because its non-flammability makes it much safer than ethanol for helicopter operations, which would be required for biological inventories over much of Alaska National Wildlife Refuges. We had tested the use of propylene glycol for samples, intended to be processed by HTS methods, in previous studies (Bowser et al. 2017, Bowser et al. 2019).

### Laboratory methods

**Figure 4.**
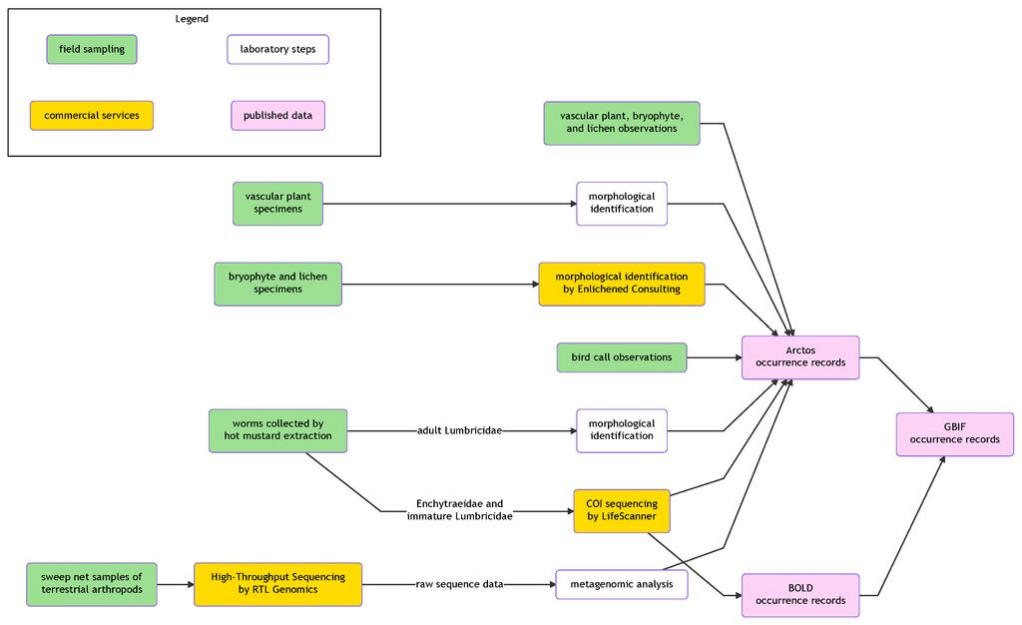
Flowchart illustrating workflows from field sampling to publication of occurrence data. The flowchart was generated using the DiagrammeR package (Iannone 2020).

Observation data and specimens were processed using methods that varied for each taxonomic group and sample type. A graphical summary of all methods used is provided in Fig. 4.

#### Plants and lichens

Vascular plants, that could not be identified in the field, were identified in the lab using pertinent keys (Hultén 1968, Welsh 1974, Tande and Lipkin 2003). Vascular plant specimens were not retained.

Lichen and bryophyte samples were sent to Trevor Goward (Enlichened Consulting Ltd., Clearwater, British Columbia) for identification. Specimens were identified by Trevor Goward and Curtis Björk (Suppl. material 3). Most specimens were discarded, but some lichen and bryophyte specimens were retained by Trevor Goward.

Plant and lichen data were entered into Arctos (https://arctosdb.org/) as observation data.

#### Bird call survey data

Bird call survey data were entered into Arctos as observation records.

#### Earthworms

Worm specimens were deposited in the Kenai National Wildlife Refuge's entomology collection*^1^, where data are managed and published through Arctos. Worm specimens were sorted to morphospecies. Adult earthworms (Lumbricidae) were identified morphologically using the key of Reynolds (1977). Representatives of each perceived morphospecies of enchytraeids and immature lumbricids in each sample were mailed to the Center for Biodiversity Genomics (Guelph, Ontario) for COI sequencing using LifeScanner kits (http://lifescanner.net).

#### Sweep net samples of terrestrial arthropods

Arthropods and any other invertebrates in the sweep net samples were separated from debris by hand under a stereomicroscope. All fragments of invertebrates were retained. Samples were stored in a -23°C freezer until they were shipped out for sequencing.

Due to budget limitations, we processed 125 of the 160 sweep net samples. We selected all 80 samples taken from the east side of each plot (40 plots × 1 sample/plot × 2 visits/plot). To choose 45 samples from the remaining 80, we selected plots spatially. First, we chose 20 samples from plots at 1 km spacing (10 plots × 2 visits/plot), then we chose 25 of 26 samples from another 13 plots that were maximally distant from these 10 plots (13 plots × 2 visits/plot). These 45 samples from west plot halves were intended to be used for estimating occupancy metrics.

Sweep net samples were shipped to RTL Genomics (http://rtlgenomics.com) for extraction and sequencing steps. DNA extraction methods are included in Suppl. material 2.

Sequencing was performed on an Illumina MiSeq platform and reads were processed using RTL Genomics’ standard methods with the *mlCOIlintF*/*HCO2198* primer set of Leray et al. (2013), yielding a 313 bp region of the COI gene. We selected this primer set because it has been shown to amplify well across a broad set of arthropod groups (Brandon-Mong et al. 2015, Hajibabaei et al. 2019).

Samples were amplified for sequencing in a two-step process. The forward primer was constructed (5’-3’) with the forward Illumina overhang adapter (TCGTCGGCAGCGTCAGATGTGTATAAGAGACAG) added to the *mlCOIlintF* primer (GGWACWGGWTGAACWGTWTAYCCYCC). The reverse primer was constructed (5’-3’) with the reverse Illumina overhang adapter (GTCTCGTGGGCTCGGAGATGTGTATAAGAGACAG) added to the *HCO2198* primer (TAAACTTCAGGGTGACCAAAAAATCA). Amplifications were performed in 25 μl reactions with Qiagen HotStar Taq master mix (Qiagen Inc, Valencia, California), 1 μl of each 5 μM primer and 1 μl of template. Reactions were performed on ABI Veriti thermocyclers (Applied Biosytems, Carlsbad, California) under the following thermal profile: 95°C for 5 min, then 35 cycles of 94°C for 30 s, 54°C for 40 s, 72°C for 1 min, followed by one cycle of 72°C for 10 min and 4°C hold.

Products from the first stage amplification were added to a second PCR, based on qualitatively determined concentrations. Primers for the second PCR were designed, based on the Illumina Nextera PCR primers as follows: Forward - AATGATACGGCGACCACCGAGATCTACAC[i5index]TCGTCGGCAGCGTC and Reverse - CAAGCAGAAGACGGCATACGAGAT[i7index]GTCTCGTGGGCTCGG. The second stage amplification was run the same as the first stage except for 10 cycles. Amplification products were visualised with eGels (Life Technologies, Grand Island, New York). Products were then pooled equimolarly and each pool was size-selected in two rounds using SPRIselect Reagent (BeckmanCoulter, Indianapolis, Indiana) in a 0.75 ratio for both rounds. Size-selected pools were then quantified using the Qubit 4 Fluorometer (Life Technologies) and loaded on an Illumina MiSeq (Illumina, Inc. San Diego, California) 2 × 300 flow cell at 10 pM.

Our metagenomic analysis was carried out on the Yeti Supercomputer (USGS Advanced Research Computing 2019). We used the SCVUC COI metabarcode pipeline (https://github.com/EcoBiomics-Zoobiome/SCVUC_COI_metabarcode_pipeline) except that we used neither the RDP Classifier (Wang et al. 2007) nor the CO1 classifier (Porter and Hajibabaei 2018a) for taxonomic assignments. We intentionally selected a metagenomics pipeline that preserved all Amplicon Sequence Variants (ASVs) for maximum reusability and comprehensiveness of the data derived from this project (see Callahan et al. 2017).

Forward and reverse reads were paired with SeqPrep (https://github.com/jstjohn/SeqPrep) using the default settings of a minimum quality of Phred of 20 and an overlap of at least 25 bp. We removed forward and reverse primers with cutadapt v2.3 (Martin 2011, http://cutadapt.readthedocs.io/en/stable/index.html), accepting default settings but requiring a minimum length after trimming of at least 150 bp, minimum read quality of Phred 20 at the ends of the sequences and allowing a maximum of 3 Ns. We de-replicated FASTA files using VSEARCH 2.4.3 (Rognes et al. 2016). We de-noised reads using USEARCH v11 (Edgar 2010) with the UNOISE3 algorithm (Edgar 2016) specifying a minimum abundance of 3. The resulting ASV table and ASV sequences are provided in Suppl. material 4 and Suppl. material 5.

We made initial taxonomic assignments to all Amplicon Sequence Variants (ASVs) using the bold_identify command of the bold package version 0.8.6 (Chamberlain 2018) in R version 3.5.1 (R Core Team 2018), using the COX1 reference dataset of BOLD (Ratnasingham and Hebert 2007). In all cases of potential new distribution records, we manually scrutinised the results of BOLD Identification Engine (http://boldsystems.org/index.php/IDS_OpenIdEngine) and NCBI Nucleotide BLAST (Altschul et al. 1990) searches.

For consistency, clarity and transparency in the use of provisional names, we followed the standards of Open Nomenclature (Sigovini et al. 2016) in assigning identifications. Amplicon Sequence Variants that could not be confidently assigned to described species were assigned to BOLD Barcode Index Numbers (BINs, Ratnasingham and Hebert 2013). Amplicon Sequence Variants that could be assigned to neither species nor BINs were given provisional names including ASV labels, e.g. "*Liriomyza* sp. SlikokOtu253".

In order to exclude potential false positive detections as defined by MacKenzie et al. (2002) and MacKenzie et al. (2005) due to demultiplexing errors, we conservatively removed from the ASV table all occurrences that represented less than 0.05% of the total number of reads for any ASV, based on assuming a 0.01% to 0.03% rate of mis-assignment of reads (Deiner et al. 2017). We also removed all occurrences represented by only one or two reads.

We removed sequences from fungi, bacteria, red algae and humans by first constructing a phylogenetic tree using qiime phylogeny align-to-tree-mafft-fasttree (Lane 1991, Price et al. 2010, Katoh and Standley 2013, Bolyen et al. 2018), then pruning off branches of non-target groups on iTOL (Letunic and Bork 2019). An interactive version of this tree is available on iToL at https://itol.embl.de/tree/1641591522370151564097588. Finally, we filtered the ASV table, based on the ASVs retained in the pruned tree.

### Data analysis

For the purposes of analyses, we considered BIN identifications to be species-resolution identifications. We also removed occurrence records greater than 200 m from plot centres, consistent with the 200 m cut-off used by Morton et al. (2009) in the analysis dataset. To make sampling effort consistent across all plots, we removed all sweep-net samples taken from the west side of plots.

Analyses and plotting were performed under R, version 3.5.1 (R Core Team 2018) using the packages DiagrammeR, version 1.0.5 (Iannone 2020); GISTools, version 0.7-4 (Brunsdon and Chen 2014); maptools, version 0.9-4 (Bivand and Lewin-Koh 2018); phytools, version 0.6-99 (Revell 2012); raster, version 2.8-4 (Hijmans 2018); recluster, version 2.8 (Dapporto et al. 2015); reshape2, version 1.4.3 (Wickham 2007); rgdal, version 1.3-6 (Bivand et al. 2018); rgeos, version 0.4-2 (Bivand and Rundel 2018); vegan, version 2.5-3 (Oksanen et al. 2018); and VennDiagram, version 1.6.20 (Chen 2018).

To compare the distribution of non-native species detected in this study to the previously known distributions of non-native species in our study area, we generated a map of non-native species records. We downloaded non-native plant occurrences from the Alaska Exotic Plants Information Clearinghouse (AKEPIC 2016). We also downloaded all occurrence records for the study area from the Global Biodiversity Information Facility (GBIF 2016) and selected names that were recognised as non-native in Alaska by Simpson et al. (2019).

The total numbers of species in each phylum were estimated using the Chao estimator (Chao 1987, Chiu et al. 2014) implemented by the specpool function of the vegan package. We generated species accumulation curves using the specaccum function from the vegan package.

To classify observed communities, we first removed all rare species that were detected on less than 5% of plots, yielding an observation matrix of 40 sites and 415 species. From this, we generated a UPGMA (unweighted pair group method with arithmetic mean, Sokal and Michener 1958) consensus tree using the recluster.cons command of the recluster package, accepting default values, except that the number of trees was set to 1,000 and the Jaccard index (Jaccard 1901) was used for calculating distances. We obtained bootstrap values for nodes of the tree with the recluster.boot command of the recluster package, generating 1,000 trees with the same parameters as the original tree.

To examine community relationships, we used Nonmetric Multidimensional Scaling (NMDS) by running the same observation matrix of 40 sites and 415 species through the metaMDS function of the vegan package. As in the clustering analysis, we used the Jaccard index as the distance measure. We set the number of dimensions to two because adding more dimensions decreased the stress only slightly. To determine community membership, all species detected at 25% or more of sites in a community were assigned to that community.

### Data publishing

We sought to follow the guidelines of Penev et al. (2017) for publication of biodiversity data. Field notebooks, field data sheets, laboratory notebooks and occurrence data have been made available via Arctos (https://arctosdb.org/) and are associated together via an Arctos project (http://arctos.database.museum/project/10002227). These occurrence data on Arctos have been published to GBIF via the VertNet IPT (http://ipt.vertnet.org/). The occurrence data used in this analysis are provided in Suppl. material 6 and Suppl. material 9.

Sequence data from worm specimens sequenced using LifeScanner kits have been made publicly available through BOLD (http://boldsystems.org/). Raw sequence data from the sweep net samples of terrestrial arthropods have been been published via Zenodo (Bowser 2019) and to GenBank's Sequence Read Archive in accessions SRR10454582–SRR10454706 under BioProject PRJNA427721.

## Results

### Occurrences

**Figure 5.**
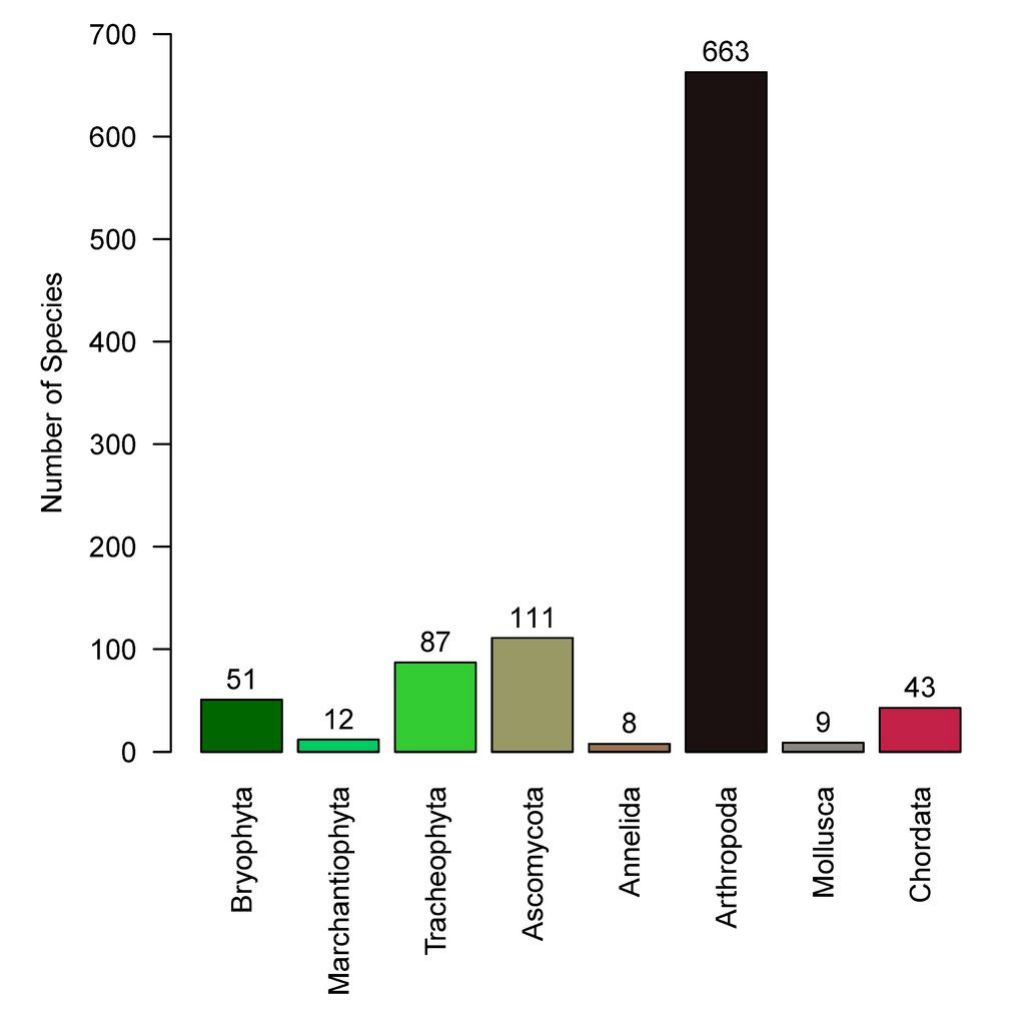
Total numbers of species observed in each phylum.

**Figure 6.**
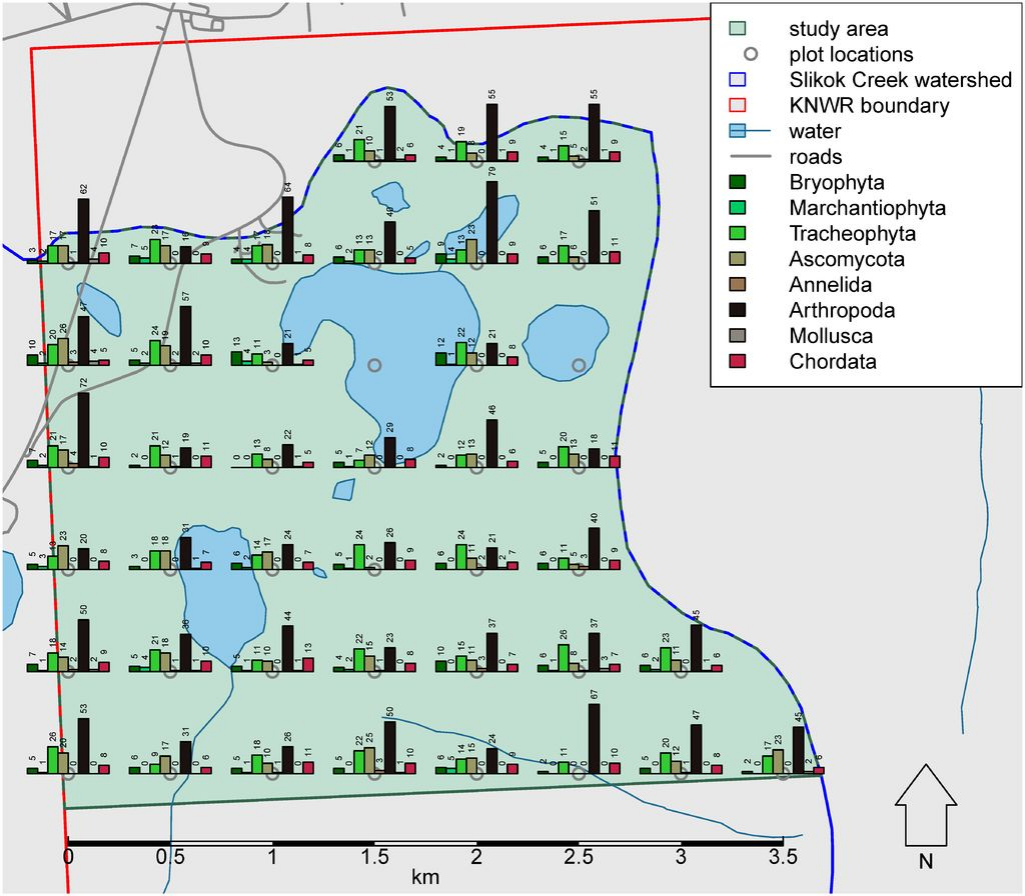
Map showing numbers of species by phyla documented at each site.

Collectively, 4,764 catalogued occurrence records were generated (Suppl. material 6), of which 4,703 were within 200 m from plot centres. A total of 710 formally described species were documented. An additional 274 ASVs were identified with BIN identifications, making a total of 984 species or BIN identifications (Fig. 5). From this point on, all numbers reported and figures include BIN identifications as "species". We documented 49 to 137 species per site (mean = 88, Fig. 6).

Of the 397 described arthropod species documented, 102 (26% of the described arthropod species found) appear to be newly reported from Alaska (Suppl. material 7). The new records included 65 Diptera, 14 Hymenoptera, 11 Hemiptera, 8 Lepidoptera, 2 Neuroptera and 1 species of Psocodea and Araneae. Five species (*Allodiaczernyi* (Landrock, 1912) (Diptera: Mycetophilidae); *Exechiaparva* Lundström, 1909 (Diptera: Mycetophilidae); *Idioceruselegans* Flor, 1861 (Hemiptera: Cicadellidae); *Rymosiapinnata* Ostroverkhova, 1979 (Diptera: Mycetophilidae); and *Zygoneurasciarina* Meigen, 1830 (Diptera: Sciaridae)) appear to be new records for North America.

**Figure 7.**
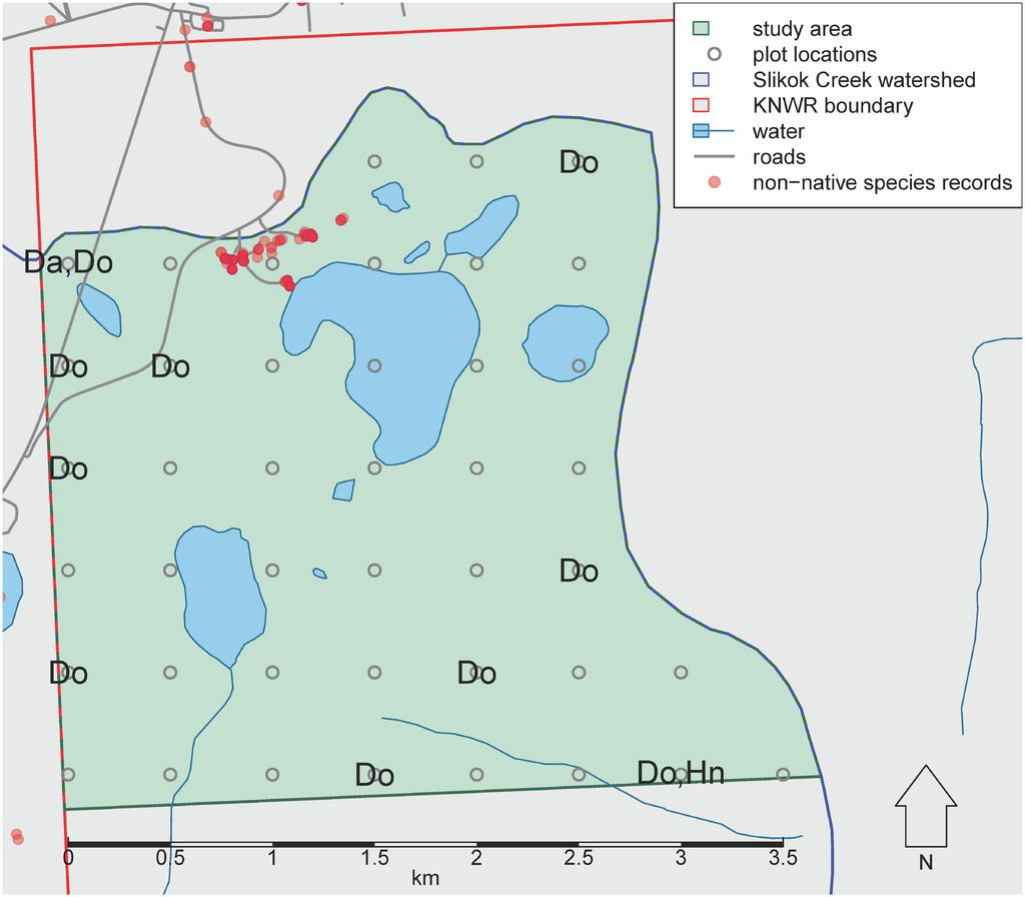
Locations where non-native species were detected. Da: *Derocerasagreste*. Do: *Dendrobaenaoctaedra*. Hn: *Heterarthrusnemoratus*. Red dots signify non-native species records from AKEPIC (2016) and GBIF (2016).

We detected three non-native species: *Derocerasagreste* (Linnaeus, 1758) (Stylommatophora: Agriolimacidae), *Dendrobaenaoctaedra* (Savigny, 1826) (Crassiclitellata: Lumbricidae) and *Heterarthrusnemoratus* (Fallén, 1808) (Hymenoptera: Tenthredinidae). *Derocerasagreste* was found at one site less than 100 m from a road, *Dendrobaenaoctaedra* was widespread over the study area and *Heterarthrusnemoratus* was found at one site more than 3 km from human development (Fig. 7).

Amongst the birds observed were three species of special interest. We documented *Sittacanadensis* Linnaeus, 1766 (Passeriformes: Sittidae) at four sites. *Regulussatrapa*, Lichtenstein, 1823 (Passeriformes: Regulidae) was detected at six sites. *Contopuscooperi* Nuttall, 1831 (Passeriformes: Tyrannidae) was documented at ten sites.

One species of potential conservation concern, *Lathrapantelesheleios* Williams, 1985 (Hymenoptera: Braconidae) was detected on two separate occasions at a single site. The COI sequence which we obtained was 98.53% similar (p-dist) to a specimen with processid JSHYO264-11, identified as *Lathrapantelesheleios* and it was placed within a clade of sequences of this species (Suppl. material 8).

### Species diversity

**Figure 8.**
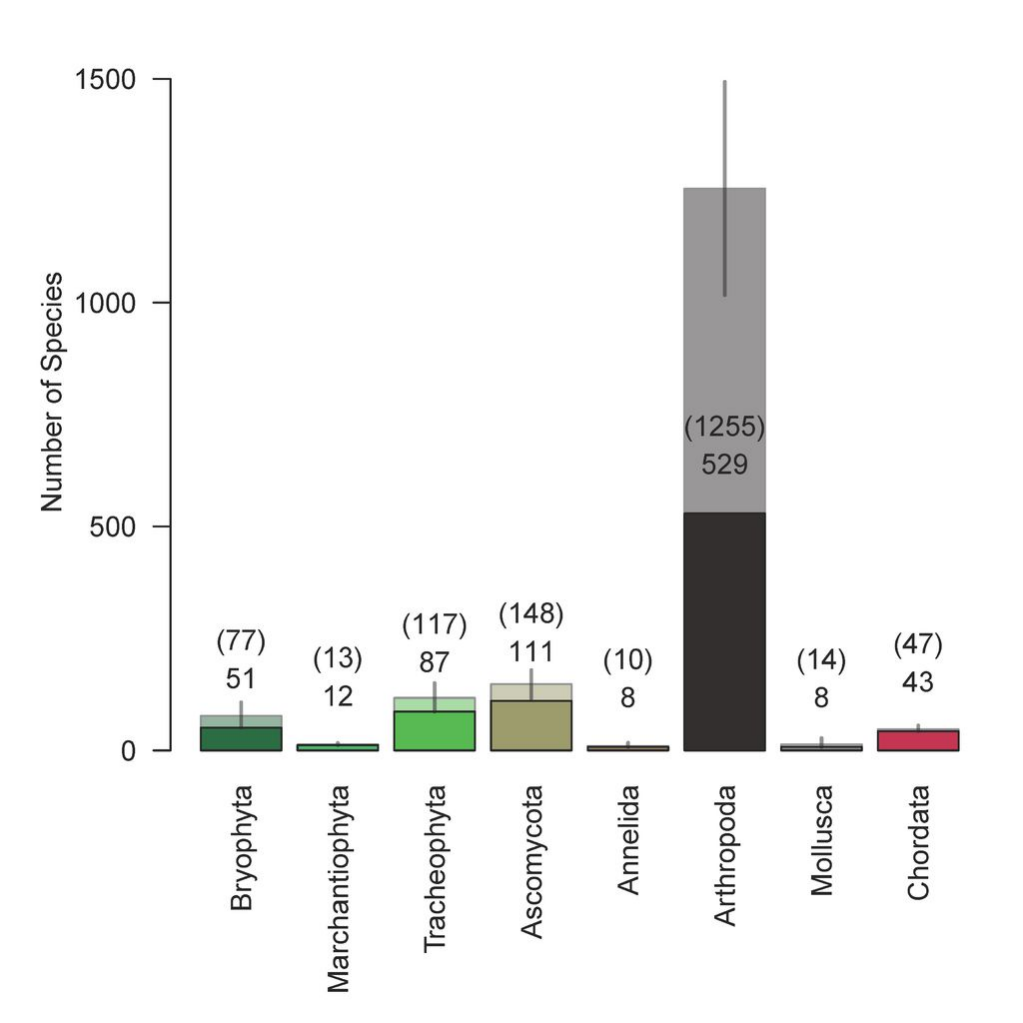
Observed and estimated numbers of species from phyla in the analysis dataset. Darker boxes and lower numbers are observed numbers of species; paler boxes and upper numbers in parentheses are Chao estimates of the total species pool. Error bars are 2× the standard errors of the Chao estimates except that the lower bounds of error bars were truncated at the observed numbers of species.

**Figure 9.**
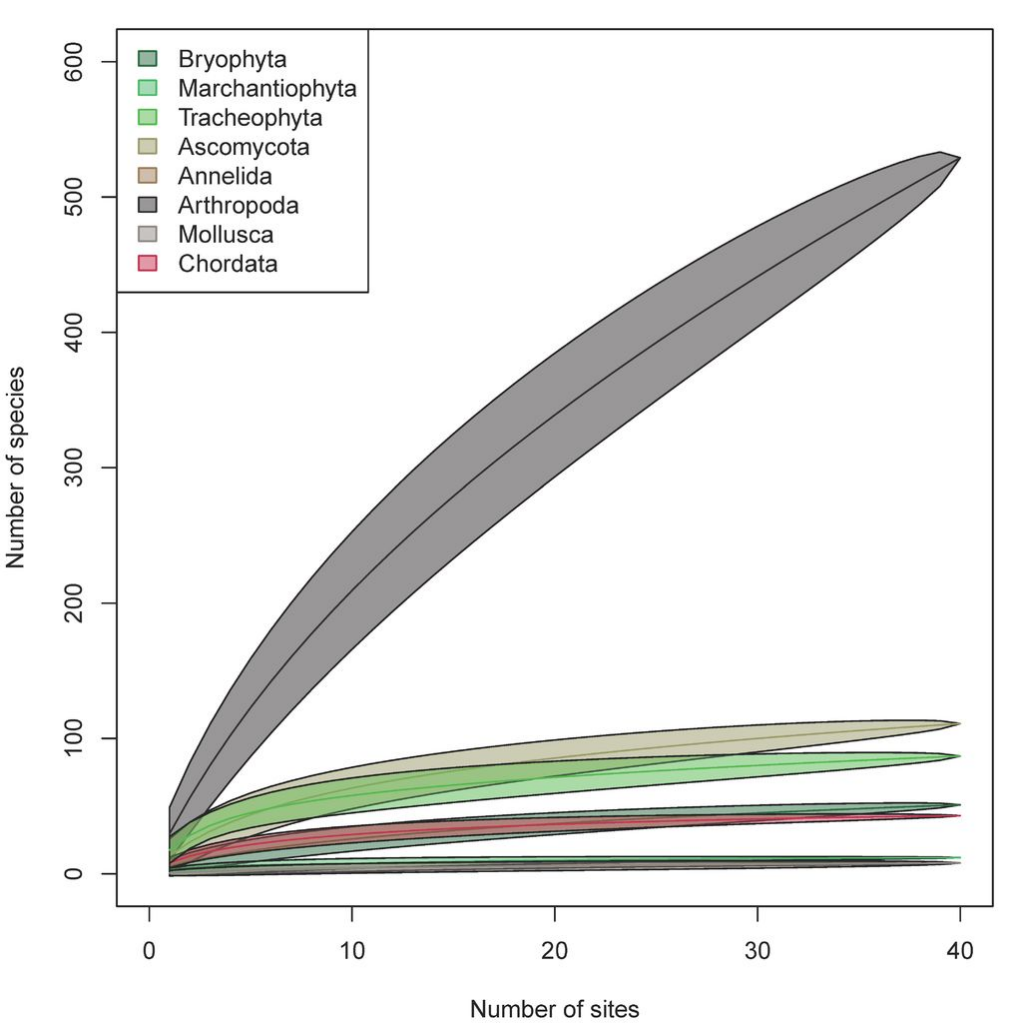
Species accumulation curves based on the analysis dataset. Centre lines represent the estimates and the polygons indicate ± 2× standard error of the estimates.

The analysis dataset (Suppl. material 9) included 3,090 occurrence records of 849 species. For all taxa except arthropods, the methods used captured more than half of the estimated numbers of species that could be detected using our methods (Table 1, Fig. 8); for arthropods, we detected only 42% of the estimated number of species. Based on species accumulation curves, adding more sites would contribute relatively few species per plot for all taxa except for arthropods (Fig. 9). The slope of the species accumulation curve at the 39^th^ site ranged from 0.05 species added per additional site for segmented worms (Annelida) and liverworts (Marchantiophyta) to 8 for arthropods (Table 1).

### Communities

**Figure 10.**
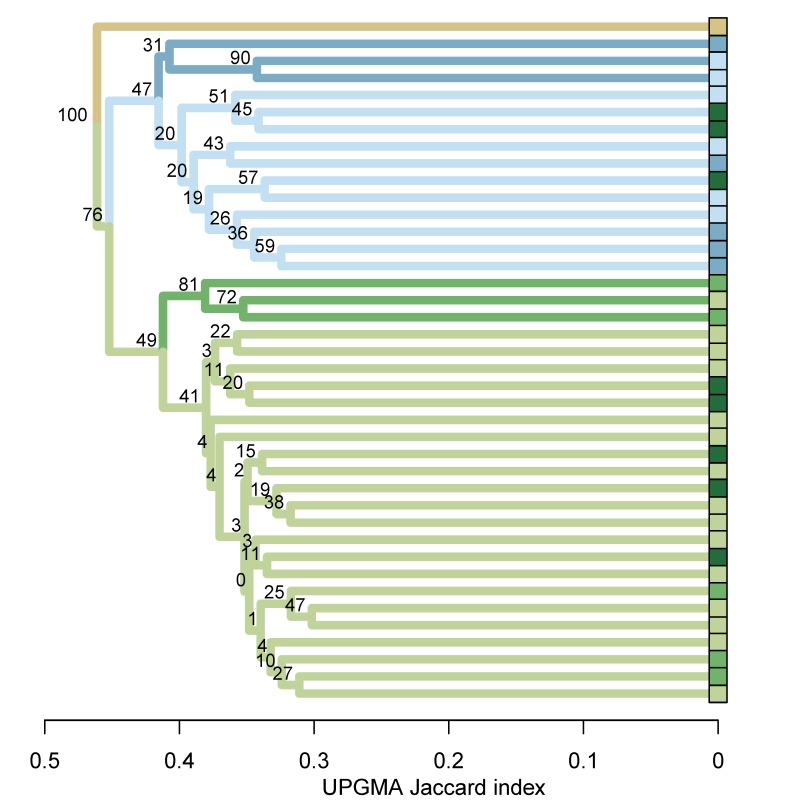
UPGMA tree of communities at sampling sites, based on Jaccard coefficients. Colour-filled boxes represent sampling sites. Colours in filled boxes correspond to colours of land cover classes from Homer et al. (2015) and Fig. 2. Five groups have been highlighted by coloured tree branches.

The UPGMA tree grouped the 40 sites into five community groups: 22 in upland mixed forest, 11 in black spruce forest, 3 in open deciduous forest, 3 in shrub-sedge bog and 1 in willow. This grouping remained consistent even when different clustering methods were used and when rare species were included. These community groupings also loosely corresponded to the NLCD classification of these sites (Fig. 10).

**Figure 11.**
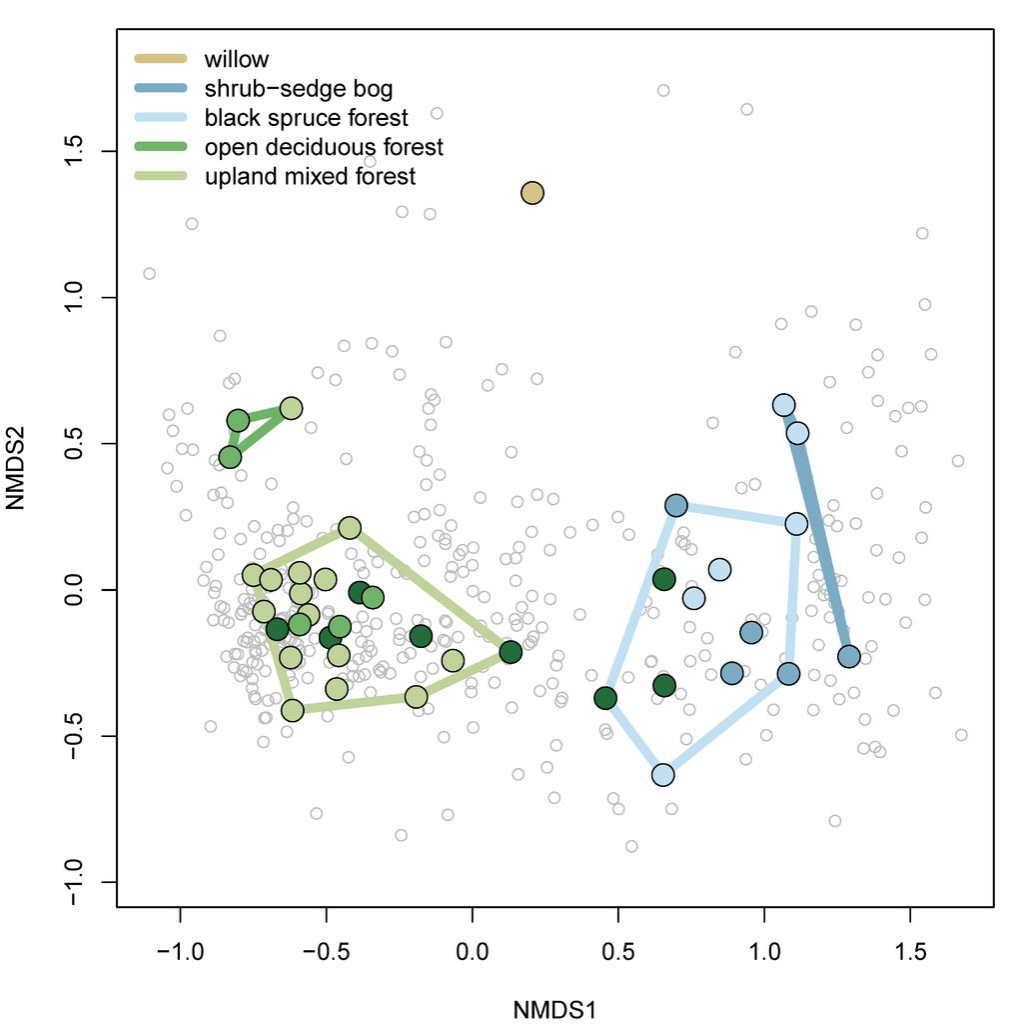
Nonmetric Multidimensional Scaling biplot, NMDS axes 1 and 2. Colours of sites (filled circles) correspond to colours of land cover classes from Homer et al. (2015) and Fig. 2. Polygons are convex hulls of the groups identified by the cluster analysis (Fig. 10). Species are represented by open, grey circles.

The NMDS analysis including two dimensions resulted in a stress value of 0.13, a "satisfactory" stress value according to the guidelines of McCune and Grace (2002). The five community groups from the cluster analysis were also separated along the NMDS axes (Fig. 11).

**Figure 12.**
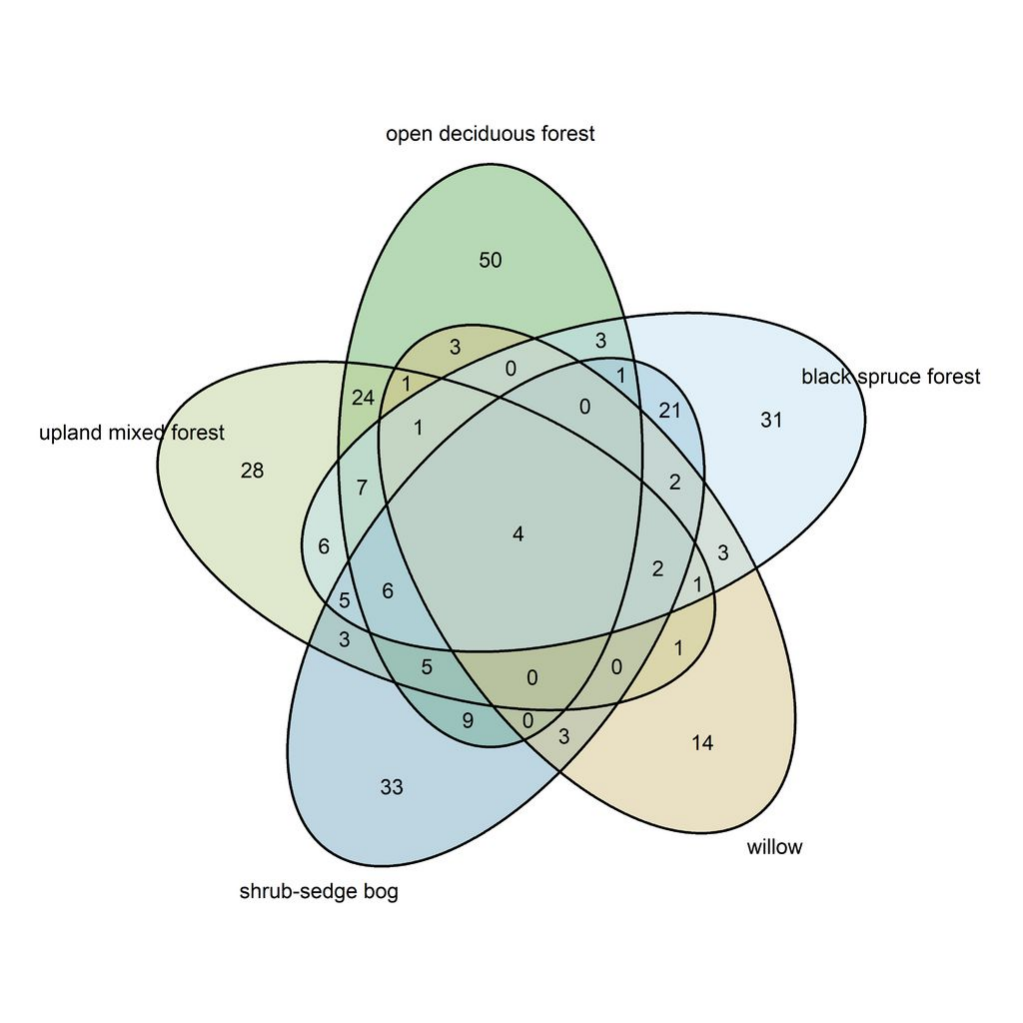
Venn diagram of species included in the community groupings.

The species included in each community are provided in Suppl. material 10. The open deciduous forest community included the highest number of species unique to that community (Fig. 12); the willow community had the fewest unique species. Four species (*Calamagrostiscanadensis* (Michx.) P.Beauv. (Poales: Poaceae), *Setophagacoronata* (Passeriformes: Parulidae), *Loxialeucoptera* (Passeriformes: Fringillidae) and *Juncohyemalis* (Passeriformes: Emberizidae)) were members of all five communities.

The open deciduous forest community (3 sites) of 114 member species was characterised by the shrubs *Alnusviridis* (Chaix) DC. (Fagales: Betulaceae) and *Oplopanaxhorridus* Miq. (Apiales: Araliaceae) under an open hardwood overstorey of *Betulaneoalaskana* Sarg. (Fagales: Betulaceae) or Populus×hastata Dode (Malpighiales: Salicaceae). Other species included *Calamagrostiscanadensis*, *Catharusustulatus* (Nuttall, 1840) (Passeriformes: Turdidae), *Dryopterisexpansa* (C.Presl) Fraser-Jenk. & Jermy (Polypodiales: Dryopteridaceae), *Empidonaxalnorum* Brewster, 1895 (Passeriformes: Tyrannidae), *Equisetumarvense* L. (Equisetales: Equisetaceae), *Fanniabrooksi* Chillcott, 1961 (Diptera: Fanniidae), *Gymnocarpiumdryopteris* Newm.(Polypodiales: Cystopteridaceae), *Parmeliasulcata* Taylor (Lecanorales: Parmeliaceae), *Poecileatricapillus* (Linnaeus, 1766) (Passeriformes: Paridae), *Setophagacoronata* and *Trientaliseuropaea* L. (Ericales: Primulaceae).

The upland mixed forest community (22 sites) of 94 species included an overstorey of *Betulaneoalaskana*, *Piceaglauca* (Moench) Voss (Pinales: Pinaceae), and *Populustremuloides* Michx. (Malpighiales: Salicaceae) with a diverse understorey of *Rosaacicularis* Lindl. (Rosales: Rosaceae), *Chamerionangustifolium* (L.) J.Holub (Myrtales: Onagraceae), *Calamagrostiscanadensis*, *Vacciniumvitis-idaea* L. (Ericales: Ericaceae), *Lycopodiumannotinum* L. (Lycopodiales: Lycopodiaceae) and *Linnaeaborealis* L. (Dipsacales: Caprifoliaceae). Additional species included *Catharusustulatus*; *Cornuscanadensis* L. (Cornales: Cornaceae); *Equisetumpratense* Ehrh. (Equisetales: Equisetaceae); *Geocaulonlividum* Fernald (Santalales: Santalaceae); *Gymnocarpiumdryopteris*; Hybotidae sp. BOLD:ACX4896 (Diptera: Hybotidae), *Hylocomiumsplendens* W.P.Schimper, 1852 (Hypnales: Hylocomiaceae); *Hypogymniaphysodes* (L.) Nyl. (Lecanorales: Parmeliaceae); *Juncohyemalis*; *Lobariapulmonaria* (L.) Hoffm. (Peltigerales: Lobariaceae); *Ochlerotatuscommunis* (De Geer, 1776) (Diptera: Culicidae); *Orthiliasecunda* (L.) House (Ericales: Ericaceae); *Parmeliasulcata*; *Pleuroziumschreberi* Mitten, 1869 (Hypnales: Hylocomiaceae); *Sanioniauncinata* Loeske, 1907 (Hypnales: Amblystegiaceae); *Setophagacoronata*; and *Trientaliseuropaea*.

The shrub-sedge bog community (3 sites) was characterised by the presence of *Andromedapolifolia* L. (Ericales: Ericaceae), *Betulaglandulosa* Michx. (Fagales: Betulaceae), *Carexrotundata* Wahlenb. (Poales:Cyperaceae), *Dictynaarundinacea* (Linnaeus, 1758) (Araneae: Dictynidae), *Eudorylas* sp. BOLD:ACZ4721 (Diptera, Pipunculidae), *Ledumpalustre* L. (Ericales: Ericaceae), *Myricagale* L. (Fagales: Myricaceae), *Passerculussandwichensis* (J.F.Gmelin, 1789) (Passeriformes: Emberizidae), and *Vacciniumoxycoccos* L. (Ericales: Ericaceae).

The single shrub site straddled a small stream where a *Salixcommutata* Bebb (Malpighiales: Salicaceae), *Salixpulchra* Cham., *Myricagale*, *Calamagrostiscanadensis* and other wetland plants grew. The full list of species at this site can be obtained by searching through Suppl. material 6 for site label "SK24".

The black spruce forest community (11 sites) was characterized by *Piceamariana* Britton, Sterns & Poggenb. (Pinales: Pinaceae), *Ledumpalustre*, *Vacciniumoxycoccos*, *Betulaglandulosa*, *Empetrumnigrum* L. (Ericales: Ericaceae), *Rubuschamaemorus* L. (Rosales: Rosaceae) and *Vacciniumvitis-idaea*. Other frequent species in black spruce forest sites were *Catharusustulatus*; *Hypogymniaoccidentalis* L.H.Pike (Lecanorales: Parmeliaceae); *Hypogymniaphysodes*; *Juncohyemalis*; *Pleuroziumschreberi*; *Reguluscalendula* (Linnaeus, 1766) (Passeriformes: Regulidae); *Sphagnumangustifolium* C.E.O.Jensen, 1896 (Sphagnales: Sphagnaceae); *Sphagnumfuscum* Klinggräff, 1872; and *Vacciniumuliginosum* L. (Ericales: Ericaceae).

## Discussion

### New distribution records

Remarkably, 26% of the described arthropod species which we documented appeared to be new records for Alaska. We believe that this is partially due to the use of HTS methods, which identified many species—especially small Diptera—that have been under-surveyed in Alaska. An extensive DNA barcode library of insects from Canada (Hebert et al. 2016) enabled identification of many Alaskan species for which DNA barcodes had not been obtained by Sikes et al. (2017).

We expect that many more arthropod species could be found even in our small study area, based on species accumulation curves, especially if rare community types were intentionally sought. The Diptera, especially smaller species, are diverse in temperate and higher latitudes, with many more species expected to be described (Hebert et al. 2016, Morinière et al. 2019). Despite a similarly high expected diversity of Diptera in Alaska, the state lacks a dedicated Diptera taxonomist and there have been few efforts to inventory the Alaskan species of Diptera.

Most of the species, newly reported for Alaska, are widespread in northern North America, so finding them in our study area was not particularly surprising. We did not consider the five species of arthropods that were apparently new to Noth America to be non-native because they may be trans-Beringian species, a well-documented distribution pattern in the flora and fauna of Alaska (Hultén 1968, Scudder 1979, Danks et al. 1997).

Our detection of *Lathrapantelesheleios*, a species previously known only from southern Ontario and considered for inclusion in Species Candidate Lists of the Committee on the Status of Endangered Wildlife in Canada (Fernandez-Triana 2014), indicates that it may be much more widespread than previously thought. Alternatively, our detection may represent a closely-related congener.

We do not consider the new distribution records presented here, based on metabarcoded DNA samples, to be as verifiable as specimen-based records. We regard the new distribution records documented here as tentative until verified by specimen-based collections. However, we applied appropriate means to filter out potential false positive occurrences and we carefully scrutinised all identifications resulting in potential new distribution records. We provided the sequence data via Zenodo (Bowser 2019), GenBank, Arctos and as supplementary material here (Suppl. materials 4, 5) so that all of our identifications can be checked.

We propose that an integrative combination of specimen-based morphological identifications (Sikes et al. 2017b), DNA barcode library building through specimen-based Sanger sequencing (Hebert et al. 2016, Morinière et al. 2019) and inventories by HTS metagenomic methods for bulk samples (Morinière et al. 2019) should be pursued on Alaskan National Wildlife Refuges to enable efficient future biomonitoring through HTS methods.

### Non-native species and changing assemblages

We did not observe any non-native plants in the sites included in this study. This suggests that non-native plants are rare in the study area, particularly beyond the immediate footprint of human disturbance.

In contrast, we found three non-native animal species within the study area. *Dendrobaenaoctaedra* had already been widely documented on the Kenai National Wildlife Refuge (Saltmarsh et al. 2016). This surface-dwelling earthworm is parthenogenic (Omodeo 1955), can be spread by vehicles (Cameron et al. 2008) and is typically amongst the first species of European earthworms to invade forests in northern North America (Hale et al. 2005, Cameron et al. 2007).

*Heterarthrusnemoratus*, a non-native sawfly that mines leaves of birches, was first collected in Alaska in 2004 (Snyder et al. 2007). By 2015, this winged species had become widespread in southern Alaska (FS-R10-FHP 2016). It is likely that nearly all birch forest on the Kenai Peninsula is inhabited by *Heterarthrusnemoratus*.

*Derocerasagreste*, a Eurasian slug and agricultural pest, has only recently been documented from Alaska. As of this writing, only two other records of this species from Alaska have been published (GBIF 2019a), both from the Kenai Peninsula and both after 2016. *Derocerasagreste* has also been intercepted at "Juneau Old Docks", Juneau, Alaska on 13 June 2016 (unpublished data from U.S. Department of Agriculture, Animal and Plant Health Inspection Service, provided by Christopher H. Secary, Alaska Department of Natural Resources). At present, it appears that *D.agreste* on the Kenai Peninsula is restricted to areas close to human development.

*Sittacandensis* has become common on the Kenai Peninsula only recently. As recently as 1959, this species was not known to occur in Southcentral Alaska (Gabrielson and Lincoln 1959). In 1968, it was considered accidental on the Kenai Moose Range (the former name of KNWR) with only 3 previously-documented sightings (U.S. Fish and Wildlife Service 1968). In 1973, it was considered to be rare in the North Gulf Coast region of Alaska (Isleib and Kessel 1973). The oldest records of *Sittacandensis* from the Kenai Peninsula available on GBIF are two observations that date from 1979; from 2015 to 2018, 595 to 855 observations per year were recorded (GBIF 2019b).

*Regulussatrapa* has become a relatively common species during the breeding season over the past 20 years on the northern portion of the Kenai Peninsula, based on Breeding Bird Surveys (Pardieck et al. 2019). Gabrielson and Lincoln (1959) described this species' distribution as occurring on the Kenai Peninsula, but highlighted the southern Kenai Peninsula habitat types similar to Kodiak Island and the coastal areas of Prince William Sound where it was common. It was listed as uncommon during the breeding season on the Kenai Moose Range (U.S. Fish and Wildlife Service 1968).

*Contopuscooperi* was identified as a priority species of concern by the Boreal Partners in Flight Working Group (Boreal Partners in Flight 2005). The global population decline of the species combined with potential threats to preferred habitat types prompted this designation. While anecdotal information indicates a continued decline of this species across the Kenai Peninsula, we detected it with regularity.

The detection of *Dendrobaenaoctaedra* and *Heterarthrusnemoratus* in parts of the Slikok watershed that are distant from obvious human disturbance means that the forest assemblage in which they were found is now a hybrid assemblage *sensu* Hobbs et al. (2009). These are two of several species that have recently become part of the hybrid assemblages of the KNWR, successfully occupying even areas that are far from human disturbance.

Other non-native species that have become widespread on KNWR within the last 100 years, but were not detected in the current study, include *Canislatrans* Say, 1823 (Thurber and Peterson 1991); *Eriocampaovata* (Linnaeus, 1760) (U.S. Fish and Wildlife Service, Region 7 2010); *Lupinuspolyphyllus* Lindl. (U.S. Fish and Wildlife Service, Region 7 2010); *Monsomapulveratum* (Retzius, 1783) (Kruse et al. 2010, U.S. Fish and Wildlife Service, Region 7 2010); *Profenusathomsoni* (Konow, 1886) (Snyder et al. 2007, FS-R10-FHP 2016); *Taraxacumofficinale* F.H.Wigg. (U.S. Fish and Wildlife Service, Region 7 2010); *Thymallusarcticus* (Pallas, 1776) (State of Alaska, Department of Fish and Game 1978); and *Trichodectescanis* (de Geer, 1778) (Schwartz et al. 1983, Wolstad 2010). Some of these species, (e.g. *Eriocampaovata*, *Monsomapulveratum*, and *Profenusathomsoni)*, were probably present in our study area, but we may have failed to detect them due to rarity of these species, patchy distribution of these sepcies, mismatch of our temporal sampling windows with the activity of these species or low probability of detection using our methods. *Taraxacumofficinale* was common in our study area, but it was mostly restricted to areas of human disturbance. *Canislatrans* was present in our study area, but we did not use methods designed to detect mammals.

### Communities

The communities which we observed fit within previous vegetation classifications in this region. Our open deciduous forest community corresponded to the I.B.2 open broadleaf forest and I.B.2.c open balsam poplar (black cottonwood) forest classes of Viereck et al. (1992) and the Alnuscrispassp.sinuata-*Echinopanaxhorridum*, B*etula papyrifera*/*Echinopanaxhorridum* and Populusbalsamiferassp.trichocarpa/*Echinopanaxhorridum* classes of DeVelice et al. (1999). Our upland mixed forest community lined up with the I.B broadleaf forest and I.C mixed forest classes of Viereck et al. (1992) and the Lutz spruce-paper birch cover types, paper birch cover types and *Picea* X *lutzii*-*Populustremuloides*/*Vacciniumvitis-idaea* class of DeVelice et al. (1999). Our shrub-sedge bog community fitted the II.C.2.j sweetgale-graminoid bog class of Viereck et al. (1992) and the *Eriophorumangustifolium*-*Trichophorumcaespitosum* and *Myricagale*/*Eriophorumangustifolium* classes of DeVelice et al. (1999). Our willow community was like the II.C.2.g willow open low scrub class of Viereck et al. (1992) and the *Myricagale*/*Calamagrostiscanadensis* class of DeVelice et al. (1999). Our black spruce forest community fitted the I.A.2.f black spruce open needleleaf forest and I.A.3.d black spruce needleleaf woodland classes of Viereck et al. (1992) and the *Piceamariana*/*Vacciniumvitis-idaea* community of Viereck et al. (1992).

### Methodological comments

While we selected a sample frame, plot sizes and other parameters carefully, generally using methods consistent with Morton et al. (2009), we recognise that these could be optimised. In particular, it would be useful to determine an optimal sweep net sample area and an optimal sequencing depth. Using a sweep net with a smaller mesh size would improve collection rates of more minute arthropods in the future (Derek Sikes, University of Alaska Museum, personal communication).

It was apparent from the species accumulation curve of arthropods that many more species remain to be collected in our study area. We believe that one reason for this pattern is low probability of detection for many species using our sweep net and metabarcoding methods.

We intentionally limited our efforts to testing methods that could efficiently be deployed over large, remote areas and deliver information on a statistically useful sample size of sampling locations. This limited the available sampling methods to active sampling methods and extraction methods. Of these, we chose sweep-net sampling because of its simplicity, because the required equipment could be compact and light, because it samples a wide diversity of insect groups (Collet 2004) and because samples could be stored and processed later. Many studies have shown sweep-net sampling to be an effective sampling method (e.g. Spafford and Lortie 2013, Shweta and Rajmohana 2018), but the weaknesses and limitations of this method are also well known. Drawbacks of the sweep net method include variation amongst collectors, differing results depending on the time of day and weather.

We recognise that other sampling methods (e.g. malaise traps) would be superior for maximising the number of species observed (Marshall et al. 1994). Morinière et al. (2019) demonstrated that malaise traps, processed by HTS methods, can be an effective method for biomonitoring Diptera. We agree with other authors that no single method samples terrestrial invertebrates exhaustively and that employing a variety of methods would deliver the most comprehensive biological inventories (Marshall et al. 1994, Yi et al. 2012).

Amongst primer pairs, differences in binding to DNA templates lead to amplification biases, affecting both read abundances and detections of species, so that any single primer set will lead to detections of a subset of species (Elbrecht and Leese 2017, Hajibabaei et al. 2019). The *mlCOIlintF*/*HCO2198* primer pair which we used amplifies well across a broad range of arthropod taxa (Leray et al. 2013, Brandon-Mong et al. 2015), but, in future efforts, we would consider selecting the *mlCOIlintF*/*jgHCO2198* primer pair, which has a more degenerate reverse primer and amplifies well across a broader range of arthropod taxa than the *mlCOIlintF*/*HCO2198* pair (Elbrecht and Leese 2017).

In future efforts, we would consider using the mBRAVE platform (http://www.mbrave.net, Ratnasingham 2019) for metabarcoding analyses. An analysis on this cloud-based platform with standardised analytical steps should be more easily repeatable than the implementation of the SCVUC COI metabarcode pipeline that we used, especially for non-specialists. Ideally, we would like to demonstrate methods that would be more accessible to non-specialists, so that metabarcoding can become more of a standard practice for biomonitoring. For beginners, Liu et al. (2019) recommended graphical-based platforms, including mBRAVE.

### Other comments

At least some of the patterns which we documented are the result of interannual variation related to cycles of the boreal forest. For example, the high frequncy of occurrence of *Loxialeucoptera* that we documented was likely related to the irruptiveness of this species. *Loxialeucoptera* is common on the Kenai Peninsula, but it is known to be erratically migratory (Gabrielson and Lincoln 1959).

## Conclusion

In the past, monitoring all but a small subset of biodiversity has been logistically and economically intractable (Dallmeier et al. 2013). However, we demonstrated practical and efficient methods that could be repeated for monitoring of a large portion of biodiversity. The combination of observation-based, specimen-based and HTS methods which we used were effective for documenting species distributions and species assemblages within the study area, although there is room for improvement, particularly in the detection of arthropod species. Biomonitoring, using such methods, could provide the kinds of data necessary for meeting the broad conservation mandates of KNWR and other Alaska National Wildlife Refuges.

### Future Directions

In future efforts, we intend to survey additional hyperdiverse portions of the terrestrial biota, including soil arthropods, soil fungi and soil bacteria, thus yielding even more complete community assemblages.

## Supplementary Material

6DDF35B9-535C-5C18-A093-F1AEE8FAE243 10.3897/BDJ.8.e50124.suppl1 Supplementary material 1 Slikok Creek watershed study area map (KMZ) Data type: geographic features
Brief description: Slikok Creek watershed study area map in Keyhole Markup Language Zipped format.
File: oo_249758.kmz https://binary.pensoft.net/file/249758 Matthew L. Bowser

3F4CC646-AD37-5DAC-B1CF-C10EC0A492EA 10.3897/BDJ.8.e50124.suppl2 Supplementary material 2 DNA extraction methods Data type: laboratory methods
Brief description: These are the DNA extraction methods that were provided by RTL Genomics used for sweep-net samples of terrestrial invertebrates.
File: oo_371050.txt https://binary.pensoft.net/file/371050 Kelli Brooks

DDEC67BA-C3A4-59BB-978A-AA3F65F69A09 10.3897/BDJ.8.e50124.suppl3 Supplementary material 3 Lichen and bryophyte identifications Data type: identifications
Brief description: This spreadsheet contains the original identifications of specimens shipped to Trevor Goward for identification. In the file, the "Arctos_GUID" field contains the original Globally Unique Identifier of the Kenai National Wildlife Refuge's herbarium samples from which the specimens came. Related data are available on-line via Arctos. For example, a sample with a GUID of KNWR:Herb:10449 is available at the URL http://arctos.database.museum/guid/KNWR:Herb:10449.
File: oo_371417.csv https://binary.pensoft.net/file/371417 Trevor Goward and Curtis Björk

E6F6EAA5-62ED-5E72-AE13-6AEE021F5848 10.3897/BDJ.8.e50124.suppl4 Supplementary material 4 ASV table Data type: ASV table
Brief description: In this spreadsheet, rows represent ASVs and columns correspond to Arctos Globally Unique Identifiers of bulk sweep-net samples.
File: oo_371431.csv https://binary.pensoft.net/file/371431 Matthew L. Bowser

1B2E15B4-E0B2-5CF9-B6F8-CF66C2F731C7 10.3897/BDJ.8.e50124.suppl5 Supplementary material 5 ASV sequences Data type: ASV sequences in FASTA format
Brief description: This file contains ASV sequences from sweep-net samples in FASTA format.
File: oo_371419.fas https://binary.pensoft.net/file/371419 Matthew L. Bowser

5E479FF8-C978-5D36-A80D-3F7D0BAB6705 10.3897/BDJ.8.e50124.suppl6 Supplementary material 6 Raw occurrence data from the Slikok watershed biotic inventory Data type: occurrences
Brief description: This dataset was downloaded from Arctos on 19 November 2019. Coordinate uncertainties vary greatly by method, ranging from 3 m for 0.25 m^2^ earthworm quadrats to hundreds of metres for birds observed on variable circular plots.
File: oo_371432.csv https://binary.pensoft.net/file/371432 Matthew L. Bowser, Rebekah Brassfield, Annie Dziergowski, Todd Eskelin, Jennifer Hester, Dawn Robin Magness, Mariah McInnis, Tracy Melvin, John M. Morton and Joel Stone

7905872F-2280-50DE-9B91-07918FFD81C4 10.3897/BDJ.8.e50124.suppl7 Supplementary material 7 Arthropod species newly reported from Alaska Data type: species checklist
File: oo_371425.csv https://binary.pensoft.net/file/371425 Matthew L. Bowser

5804D3B7-70C0-52A3-9E42-D455627996ED 10.3897/BDJ.8.e50124.suppl8 Supplementary material 8 BOLD TaxonID Tree for SlikokOtu1170 Data type: phylogenetic tree
Brief description: BOLD TaxonID Tree for SlikokOtu1170 generated by BOLD's Identification Engine, 26.December.2019
File: oo_368119.pdf https://binary.pensoft.net/file/368119 Matthew L. Bowser

DD44D067-15CE-5B23-96A7-C1DB251ACAB0 10.3897/BDJ.8.e50124.suppl9 Supplementary material 9 Analysis dataset Data type: occurrences
Brief description: This is the derived dataset created for the purpose of analysis. It includes only species-resolution identifications and only the 80 sweep-net samples taken from the east half of each plot.
File: oo_371428.csv https://binary.pensoft.net/file/371428 Matthew L. Bowser, Rebekah Brassfield, Annie Dziergowski, Todd Eskelin, Jennifer Hester, Dawn Robin Magness, Mariah McInnis, Tracy Melvin, John M. Morton and Joel Stone

C73C2D79-26F4-5546-B858-0656D6EBEC5F 10.3897/BDJ.8.e50124.suppl10 Supplementary material 10 Community composition Data type: species lists
Brief description: This file lists the species assigned to each of five community types.
File: oo_371430.csv https://binary.pensoft.net/file/371430 Matthew L. Bowser

## Figures and Tables

**Table 1.** Observed and estimated numbers of species by phyla. Chao: Chao estimator. SE: estimate of the standard error of the chao estimate. Percent observed: percentage of species observed based on the Chao estimate. Slope: the number of species added per plot at the 39^th^ plot.

Phylum	Observed	Chao	SE	Percent observed	Slope
Annelida	8	10	4	80%	0.05
Arthropoda	529	1255	119	42%	8.35
Ascomycota	111	148	16	75%	0.99
Bryophyta	51	77	15	66%	0.56
Chordata	43	47	4	91%	0.20
Marchantiophyta	12	13	2	92%	0.05
Mollusca	8	14	7	58%	0.10
Tracheophyta	87	117	16	74%	0.63

## Footnotes

*1 Kenai National Wildlife Refuge Entomology Collection:
https://www.fws.gov/refuge/Kenai/what_we_do/science/specimens.html

http://arctos.database.museum/knwr_ento

https://www.gbif.org/dataset/cb0eb5bf-4759-4aa1-92b5-4143b0a64e15
