# Supplementary material for: Towards conserving natural diversity: A biotic inventory by observations, specimens, DNA barcoding and high-throughput sequencing methods
Source: Biodivers Data J. 2020 Feb 27;8:e50124. doi: 10.3897/BDJ.8.e50124 (PMC7058680; doi:10.3897/BDJ.8.e50124)
Supplement: Supplementary material 8 — BOLD TaxonID Tree for SlikokOtu1170 [file bdj-08-e50124-s008.pdf]

BOLD TaxonID Tree

Title	:	COI FULL DATABASE includes records without species designati...
Date	:	26-December-2019
Data Type	:	Nucleotide
Distance Model	:	Kimura 2 Parameter
Marker	:	COI-5P
Codon Positions	:	1st, 2nd, 3rd
Labels	:	Extra Info, Country & Province, Family
Filters	:	Length > 200
Attachment	:	Photographs & Spreadsheet

Sequence Count	:	100
Species count	:	8
Genus count	:	4
Family count	:	1
Unidentified	:	51

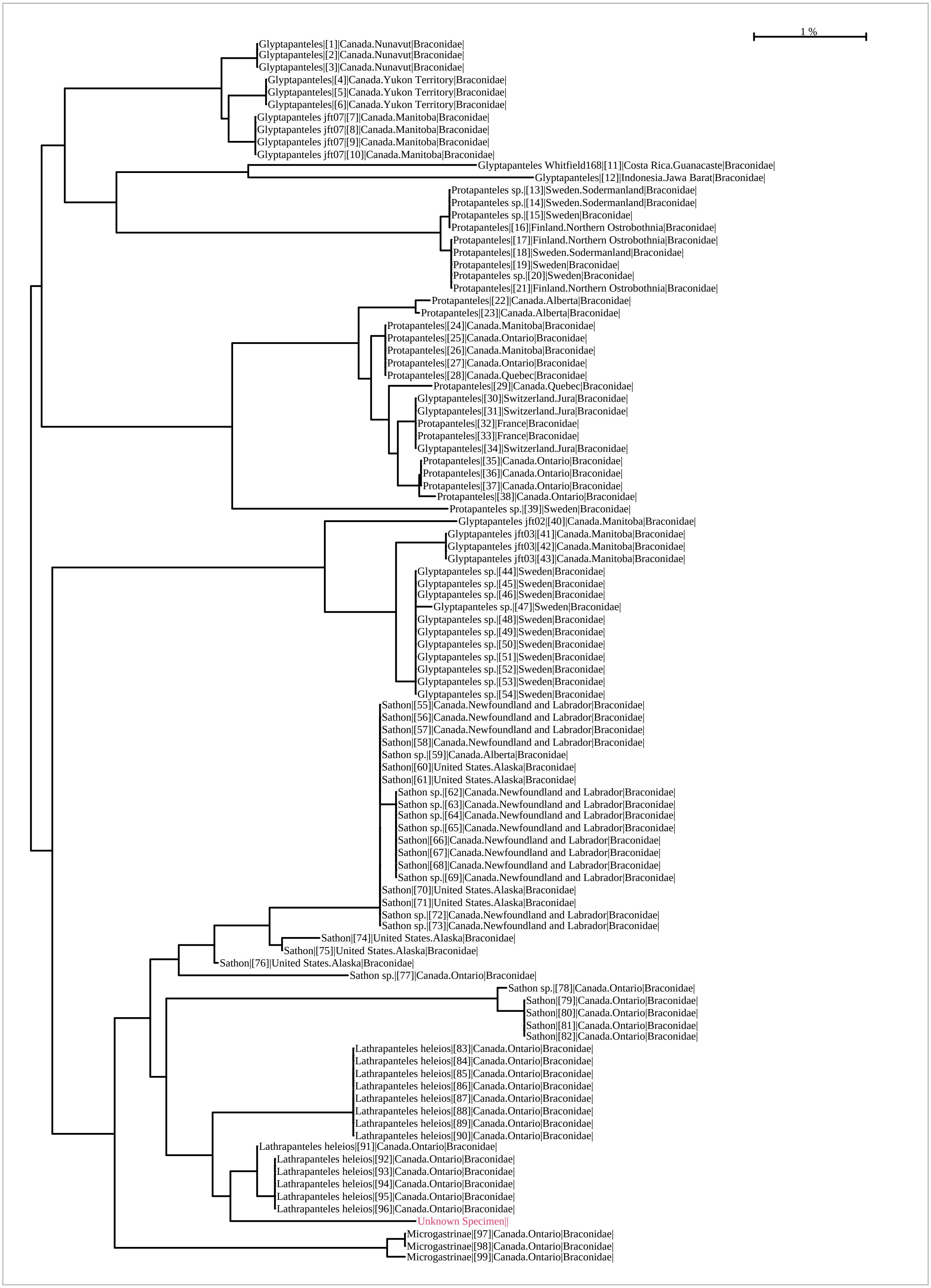
1 %
Glyptapanteles|[1]|Canada.Nunavut|Braconidae|
Glyptapanteles|[2]|Canada.Nunavut|Braconidae|
Glyptapanteles|[3]|Canada.Nunavut|Braconidae|
Glyptapanteles|[4]|Canada.Yukon Territory|Braconidae|
Glyptapanteles|[5]|Canada.Yukon Territory|Braconidae|
Glyptapanteles|[6]|Canada.Yukon Territory|Braconidae|
Glyptapanteles jft07|[7]|Canada.Manitoba|Braconidae|
Glyptapanteles jft07|[8]|Canada.Manitoba|Braconidae|
Glyptapanteles jft07|[9]|Canada.Manitoba|Braconidae|
Glyptapanteles jft07|[10]|Canada.Manitoba|Braconidae|
Glyptapanteles Whitfield168|[11]|Costa Rica.Guanacaste|Braconidae|
Glyptapanteles|[12]|Indonesia.Jawa Barat|Braconidae|
Protapanteles sp.|[13]|Sweden.Sodermanland|Braconidae|
Protapanteles sp.|[14]|Sweden.Sodermanland|Braconidae|
Protapanteles sp.|[15]|Sweden|Braconidae|
Protapanteles|[16]|Finland.Northern Ostrobothnia|Braconidae|
Protapanteles|[17]|Finland.Northern Ostrobothnia|Braconidae|
Protapanteles|[18]|Sweden.Sodermanland|Braconidae|
Protapanteles|[19]|Sweden|Braconidae|
Protapanteles sp.|[20]|Sweden|Braconidae|
Protapanteles|[21]|Finland.Northern Ostrobothnia|Braconidae|
Protapanteles|[22]|Canada.Alberta|Braconidae|
Protapanteles|[23]|Canada.Alberta|Braconidae|
Protapanteles|[24]|Canada.Manitoba|Braconidae|
Protapanteles|[25]|Canada.Ontario|Braconidae|
Protapanteles|[26]|Canada.Manitoba|Braconidae|
Protapanteles|[27]|Canada.Ontario|Braconidae|
Protapanteles|[28]|Canada.Quebec|Braconidae|
Protapanteles|[29]|Canada.Quebec|Braconidae|
Glyptapanteles|[30]|Switzerland.Jura|Braconidae|
Glyptapanteles|[31]|Switzerland.Jura|Braconidae|
Protapanteles|[32]|France|Braconidae|
Protapanteles|[33]|France|Braconidae|
Glyptapanteles|[34]|Switzerland.Jura|Braconidae|
Protapanteles|[35]|Canada.Ontario|Braconidae|
Protapanteles|[36]|Canada.Ontario|Braconidae|
Protapanteles|[37]|Canada.Ontario|Braconidae|
Protapanteles|[38]|Canada.Ontario|Braconidae|
Protapanteles sp.|[39]|Sweden|Braconidae|
Glyptapanteles jft02|[40]|Canada.Manitoba|Braconidae|
Glyptapanteles jft03|[41]|Canada.Manitoba|Braconidae|
Glyptapanteles jft03|[42]|Canada.Manitoba|Braconidae|
Glyptapanteles jft03|[43]|Canada.Manitoba|Braconidae|
Glyptapanteles sp.|[44]|Sweden|Braconidae|
Glyptapanteles sp.|[45]|Sweden|Braconidae|
Glyptapanteles sp.|[46]|Sweden|Braconidae|
Glyptapanteles sp.|[47]|Sweden|Braconidae|
Glyptapanteles sp.|[48]|Sweden|Braconidae|
Glyptapanteles sp.|[49]|Sweden|Braconidae|
Glyptapanteles sp.|[50]|Sweden|Braconidae|
Glyptapanteles sp.|[51]|Sweden|Braconidae|
Glyptapanteles sp.|[52]|Sweden|Braconidae|
Glyptapanteles sp.|[53]|Sweden|Braconidae|
Glyptapanteles sp.|[54]|Sweden|Braconidae|
Sathon|[55]|Canada.Newfoundland and Labrador|Braconidae|
Sathon|[56]|Canada.Newfoundland and Labrador|Braconidae|
Sathon|[57]|Canada.Newfoundland and Labrador|Braconidae|
Sathon|[58]|Canada.Newfoundland and Labrador|Braconidae|
Sathon sp.|[59]|Canada.Alberta|Braconidae|
Sathon|[60]|United States.Alaska|Braconidae|
Sathon|[61]|United States.Alaska|Braconidae|
Sathon sp.|[62]|Canada.Newfoundland and Labrador|Braconidae|
Sathon sp.|[63]|Canada.Newfoundland and Labrador|Braconidae|
Sathon sp.|[64]|Canada.Newfoundland and Labrador|Braconidae|
Sathon sp.|[65]|Canada.Newfoundland and Labrador|Braconidae|
Sathon|[66]|Canada.Newfoundland and Labrador|Braconidae|
Sathon|[67]|Canada.Newfoundland and Labrador|Braconidae|
Sathon|[68]|Canada.Newfoundland and Labrador|Braconidae|
Sathon sp.|[69]|Canada.Newfoundland and Labrador|Braconidae|
Sathon|[70]|United States.Alaska|Braconidae|
Sathon|[71]|United States.Alaska|Braconidae|
Sathon sp.|[72]|Canada.Newfoundland and Labrador|Braconidae|
Sathon sp.|[73]|Canada.Newfoundland and Labrador|Braconidae|
Sathon|[74]|United States.Alaska|Braconidae|
Sathon|[75]|United States.Alaska|Braconidae|
Sathon|[76]|United States.Alaska|Braconidae|
Sathon sp.|[77]|Canada.Ontario|Braconidae|
Sathon sp.|[78]|Canada.Ontario|Braconidae|
Sathon|[79]|Canada.Ontario|Braconidae|
Sathon|[80]|Canada.Ontario|Braconidae|
Sathon|[81]|Canada.Ontario|Braconidae|
Sathon|[82]|Canada.Ontario|Braconidae|
Lathrapanteles heleios|[83]|Canada.Ontario|Braconidae|
Lathrapanteles heleios|[84]|Canada.Ontario|Braconidae|
Lathrapanteles heleios|[85]|Canada.Ontario|Braconidae|
Lathrapanteles heleios|[86]|Canada.Ontario|Braconidae|
Lathrapanteles heleios|[87]|Canada.Ontario|Braconidae|
Lathrapanteles heleios|[88]|Canada.Ontario|Braconidae|
Lathrapanteles heleios|[89]|Canada.Ontario|Braconidae|
Lathrapanteles heleios|[90]|Canada.Ontario|Braconidae|
Lathrapanteles heleios|[91]|Canada.Ontario|Braconidae|
Lathrapanteles heleios|[92]|Canada.Ontario|Braconidae|
Lathrapanteles heleios|[93]|Canada.Ontario|Braconidae|
Lathrapanteles heleios|[94]|Canada.Ontario|Braconidae|
Lathrapanteles heleios|[95]|Canada.Ontario|Braconidae|
Lathrapanteles heleios|[96]|Canada.Ontario|Braconidae|
Unknown Specimen||
Microgastrinae|[97]|Canada.Ontario|Braconidae|
Microgastrinae|[98]|Canada.Ontario|Braconidae|
Microgastrinae|[99]|Canada.Ontario|Braconidae|
